# Supplementary figures and images for: High temperature environment reduces olive oil yield and quality
Source: PLoS One. 2020 Apr 23;15(4):e0231956. doi: 10.1371/journal.pone.0231956 (PMC7179852; doi:10.1371/journal.pone.0231956)

## Slide 1
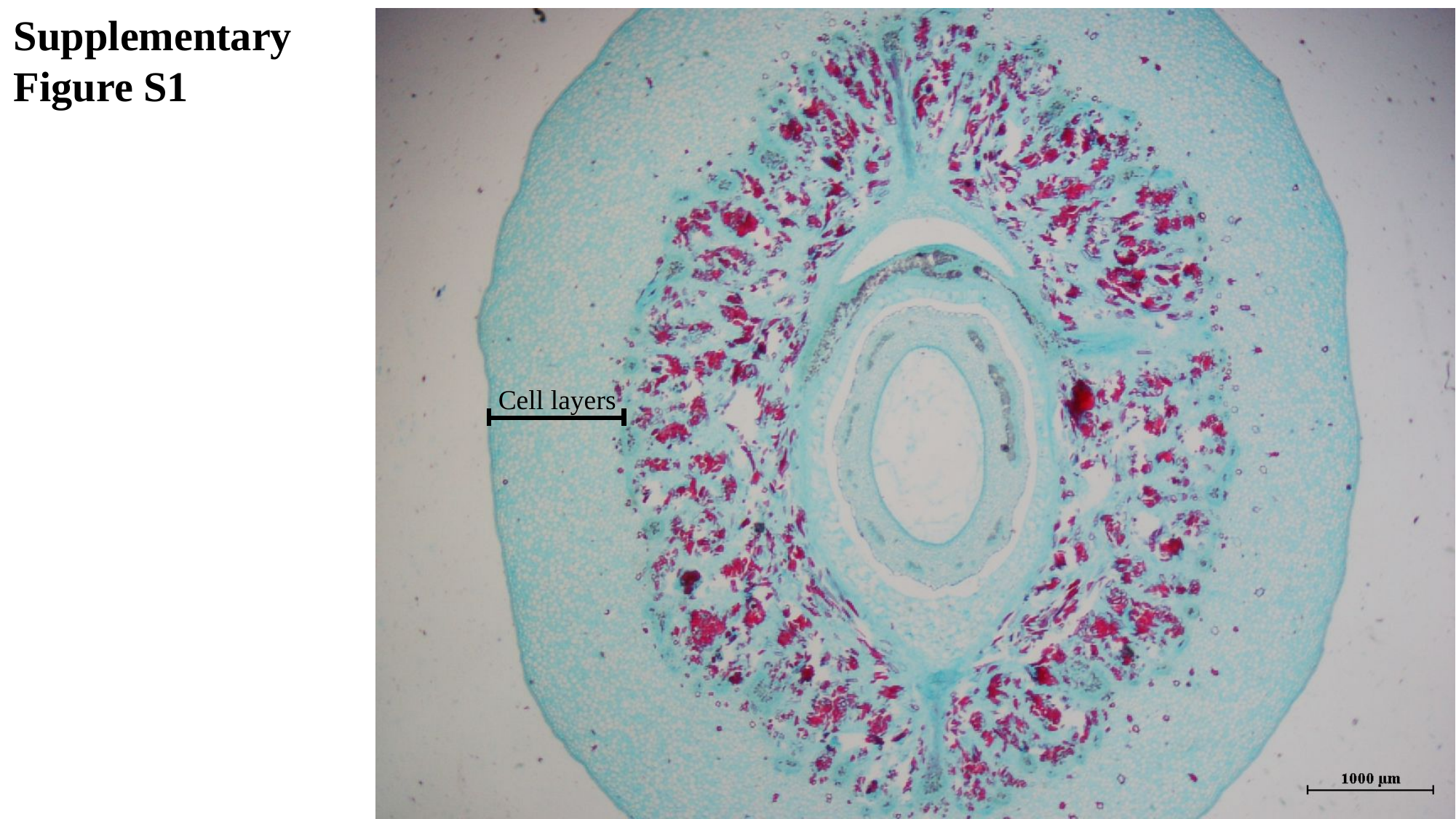

Supplementary Figure S1
Cell layers

Supplement: S1 Fig — The region of the mesocarp cell layers counted is marked. (PPTX) [file pone.0231956.s001.pptx]

## Slide 1
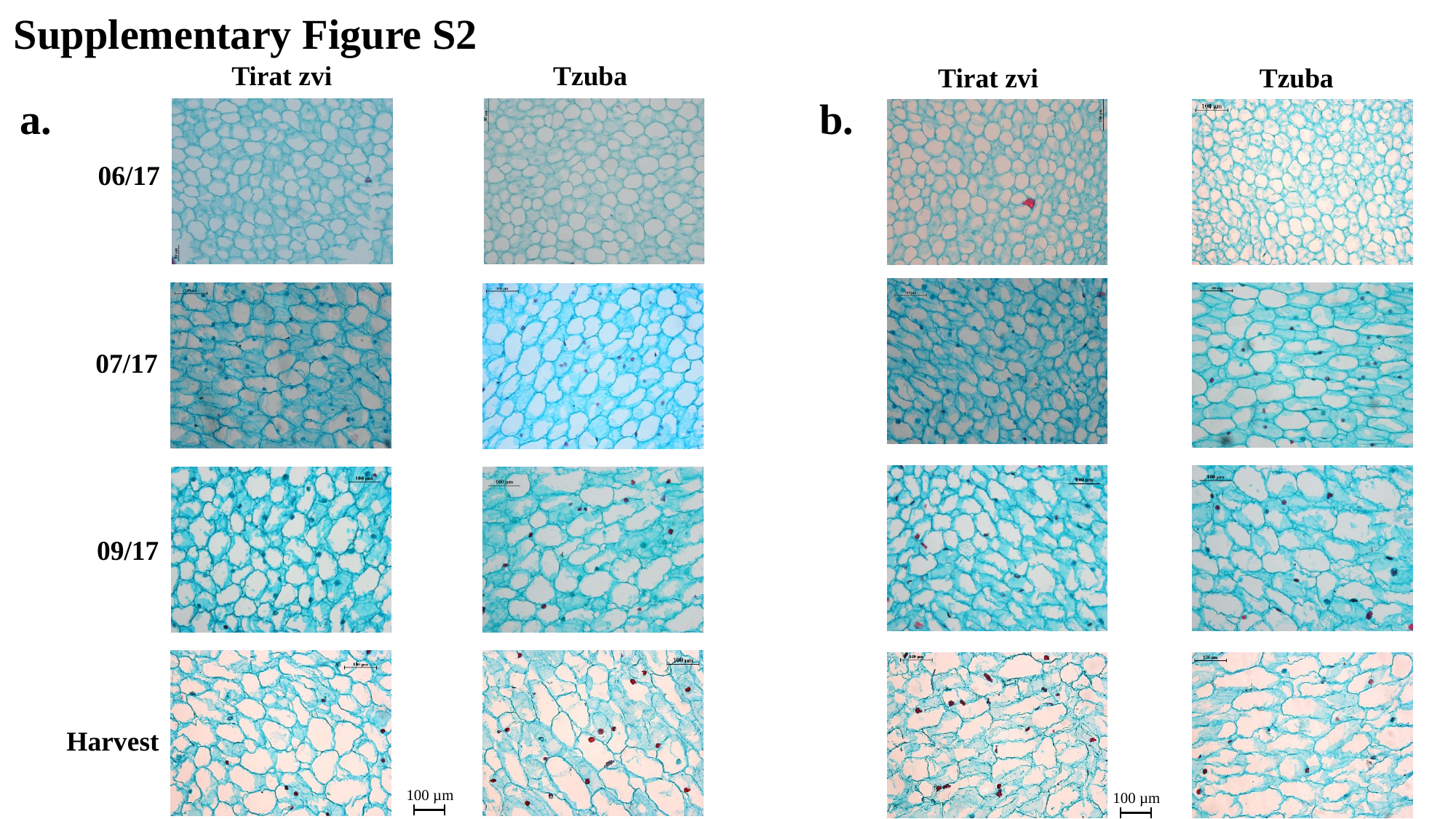

Supplementary Figure S2
Tirat zvi
Tzuba
Tirat zvi
Tzuba
a.
b.
06/17
07/17
09/17
Harvest
100 µm
100 µm

Supplement: S2 Fig — Microscope images of the mesocarp cells of 'Barnea' (a) and 'Koroneiki' (b) sampled in June, July, September and at harvest-time of the 2017 season from Tirat Zvi (HT site) and Tzuba (MT site). (PPTX) [file pone.0231956.s002.pptx]

## Slide 1
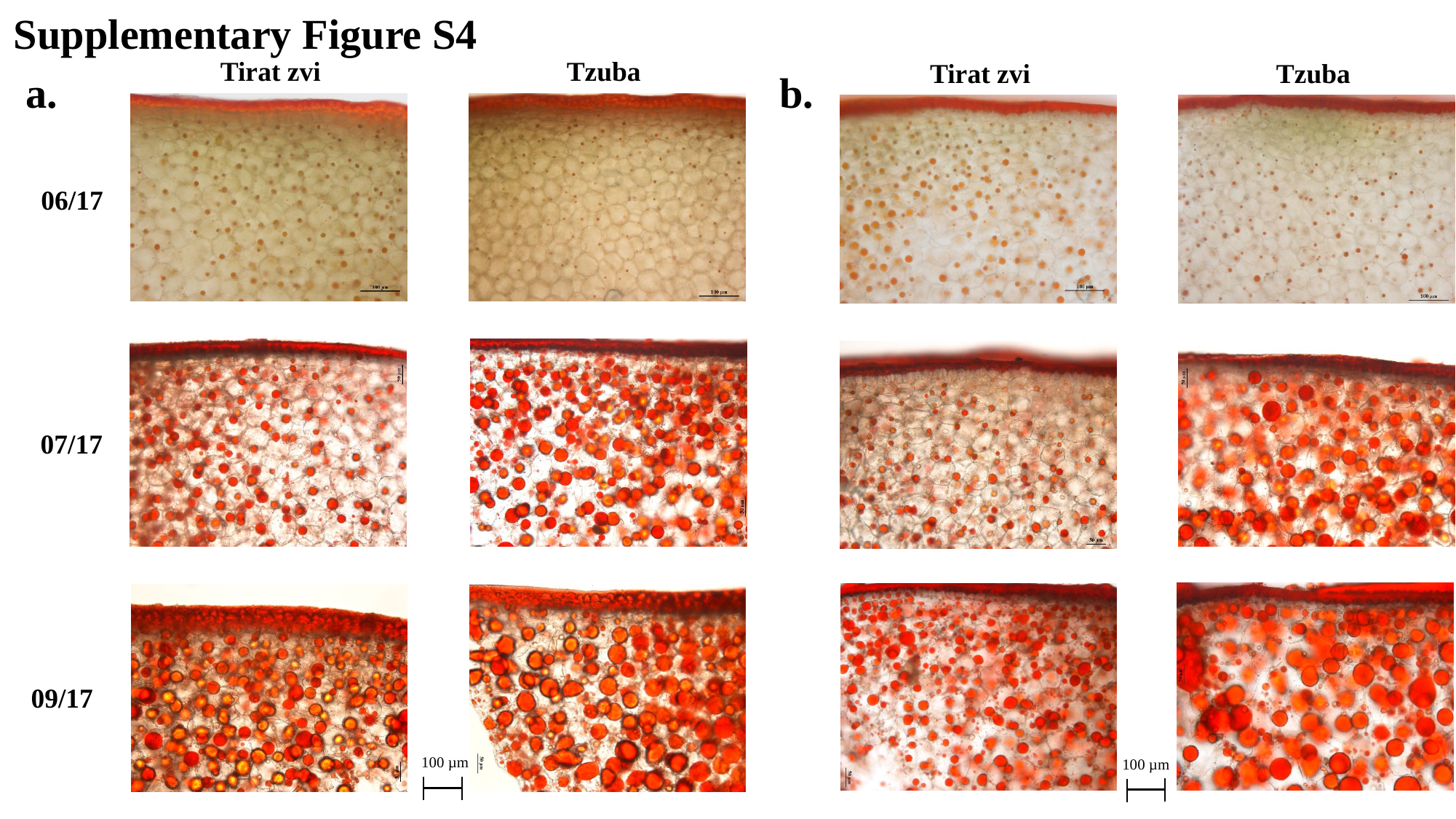

Supplementary Figure S4
Tirat zvi
Tzuba
Tirat zvi
Tzuba
a.
b.
06/17
07/17
09/17
100 µm
100 µm

Supplement: S4 Fig — Oil accumulation in 'Barnea' (a) and 'Koroneiki' (b) cultivars during 2017. Microscope images of the mesocarp cells sampled in June, July and September of 2017 season from Tirat Zvi (HT site) and Tzuba (MT site). (PPTX) [file pone.0231956.s004.pptx]
