# Supplementary material for: High temperature environment reduces olive oil yield and quality
Source: PLoS One. 2020 Apr 23;15(4):e0231956. doi: 10.1371/journal.pone.0231956 (PMC7179852; doi:10.1371/journal.pone.0231956)
Supplement: S3 Fig — Microscope images of the mesocarp cells sampled in September are presented at the left with quantification of the average cell area and the number of cell layer as well as fruit weight at the time, are presented at the right. Error bars represent confidence limits (α = 0.05). Asterisks represent significant difference (α = 0.05). (PPTX) [file pone.0231956.s003.pptx]

## Slide 1
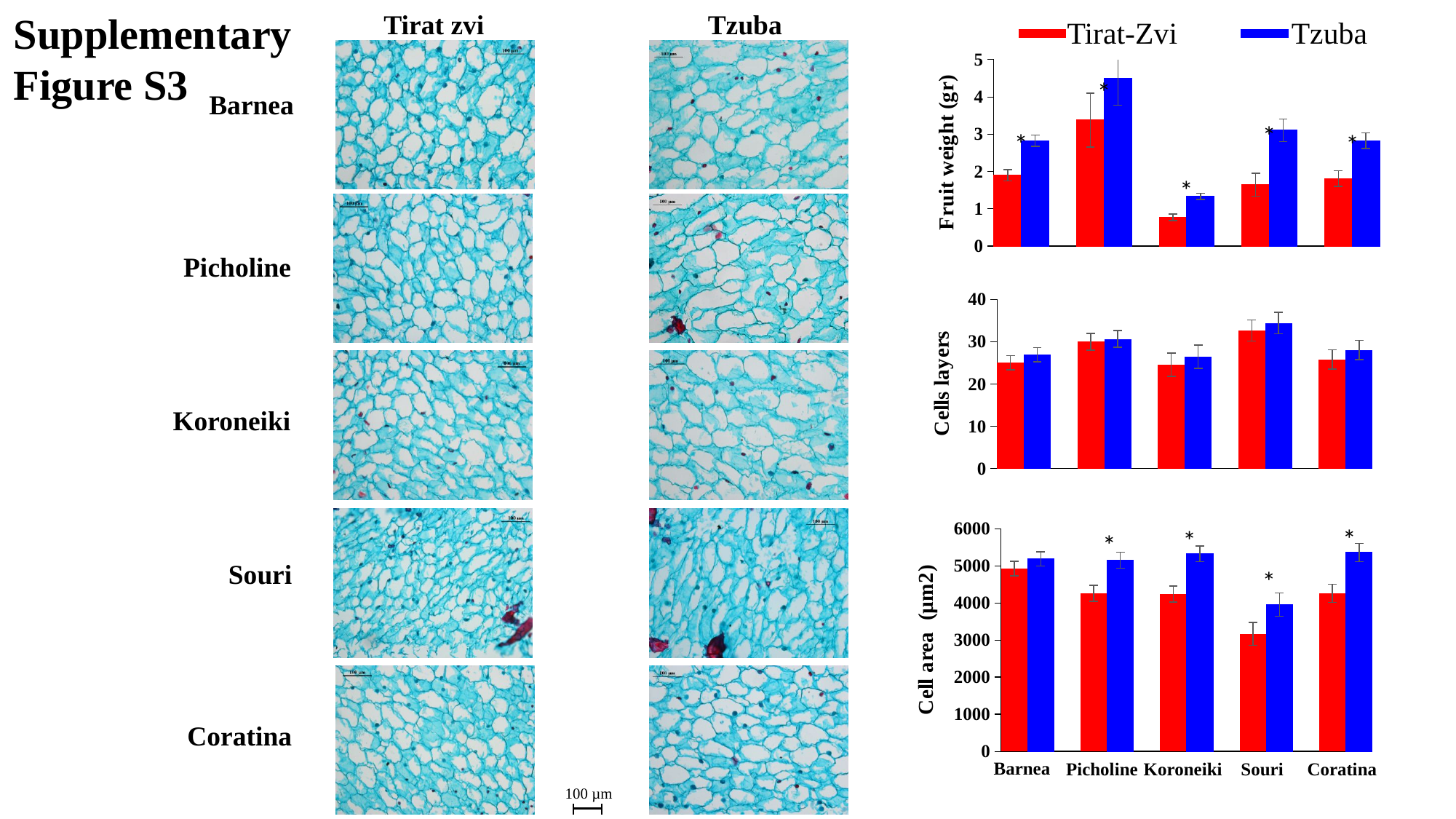

Supplementary Figure S3
Tirat zvi
Tzuba
Tirat-Zvi Tzuba
### Chart
| Category | |
|---|---|
| Barnea_Tirat Zvi | 1.8986666666666665 |
| Barnea_Tzuba | 2.828 |
| | None |
| Picholine_Tirat Zvi | 3.3770000000000002 |
| Picholine_Tzuba | 4.4990000000000006 |
| | None |
| Koroneiki_Tirat Zvi | 0.7726666666666668 |
| Koroneiki_Tzuba | 1.3325 |
| | None |
| Souri_Tirat Zvi | 1.6446666666666667 |
| Souri_Tzuba | 3.103 |
| | None |
| Coratina_Tirat Zvi | 1.8086666666666666 |
| Coratina_Tzuba | 2.823 |*
Barnea
*
*
*
*
Picholine
### Chart
| Category | |
|---|---|
| Barnea | 25.0299 |
| | 26.9046 |
| | None |
| Picholine | 30.0 |
| | 30.6183 |
| | None |
| Koroneiki | 24.5447 |
| | 26.4386 |
| | None |
| Souri | 32.6392 |
| | 34.3959 |
| | None |
| Coratina | 25.8266 |
Koroneiki
### Chart
| Category | |
|---|---|
| Barnea | 4920.8 |
| | 5181.219999999999 |
| | None |
| Picholine | 4255.73 |
| | 5148.599999999999 |
| | None |
| Koroneiki | 4238.990000000001 |
| | 5318.42 |
| | None |
| Souri | 3162.2400000000002 |
| | 3951.65 |
| | None |
| Coratina | 4258.11 |
*
*
*
Souri
*
Coratina
Barnea
Souri
Coratina
Picholine
Koroneiki
100 µm
