# Supplementary material for: High temperature environment reduces olive oil yield and quality
Source: PLoS One. 2020 Apr 23;15(4):e0231956. doi: 10.1371/journal.pone.0231956 (PMC7179852; doi:10.1371/journal.pone.0231956)
Supplement: S5 Fig — Microscope images of oil drops in fruits sampled at September are presented at the left and quantification of the average oil drop area, the density of oil drops as well as the oil content in September 2017 are presented at the right. Error bars represent confidence limits (α = 0.05). Asterisks represent significant difference (α = 0.05). (PPTX) [file pone.0231956.s005.pptx]

## Slide 1
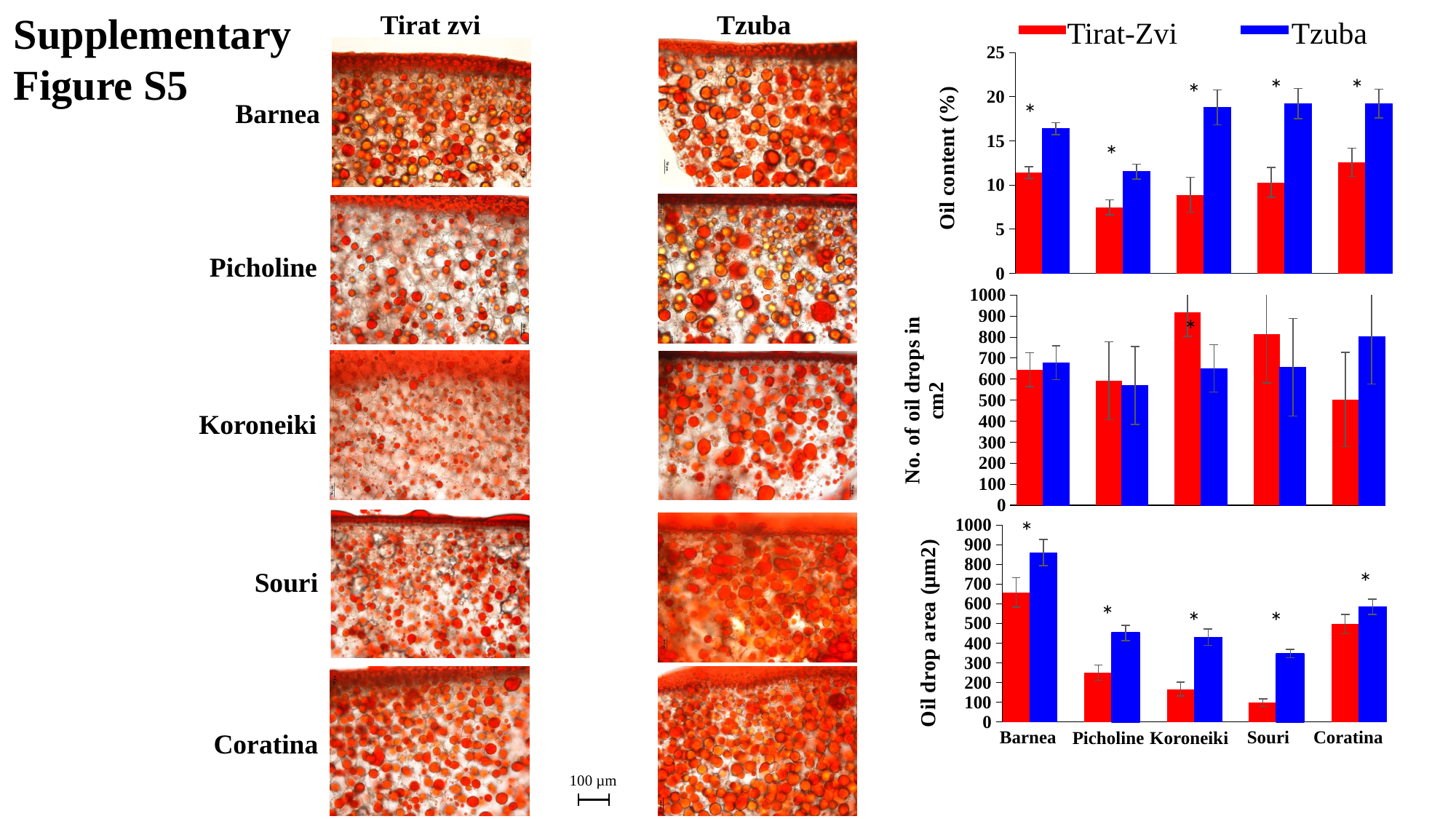

Supplementary Figure S5
Tirat zvi
Tzuba
Tirat-Zvi Tzuba
### Chart
| Category | |
|---|---|
| Barnea_Tirat Zvi | 11.404864639349922 |
| Barnea_Tzuba | 16.381104129142606 |
| | 0.0 |
| Picholine_Tirat Zvi | 7.480325956031001 |
| Picholine_Tzuba | 11.509375589591354 |
| | 0.0 |
| Koroneiki_Tirat Zvi | 8.889996339299376 |
| Koroneiki_Tzuba | 18.783543209511585 |
| | 0.0 |
| Souri_Tirat Zvi | 10.287863750792805 |
| Souri_Tzuba | 19.230730603249192 |
| | 0.0 |
| Coratina_Tirat Zvi | 12.58188669752571 |
| Coratina_Tzuba | 19.22199328165608 |
*
*
*
Barnea
*
*
Picholine
### Chart
| Category | |
|---|---|
| Barnea_Tirat Zvi | 645.439 |
| Barnea_Tzuba | 677.711 |
| | None |
| Picholine_Tirat Zvi | 591.652 |
| Picholine_Tzuba | 570.138 |
| | None |
| Koroneiki_Tirat Zvi | 917.061 |
| Koroneiki_Tzuba | 650.818 |
| | None |
| Souri_Tirat Zvi | 814.867 |
| Souri_Tzuba | 656.196 |
| | None |
| Coratina_Tirat Zvi | 502.904 |
| Coratina_Tzuba | 801.42 |*
Koroneiki
### Chart
| Category | |
|---|---|
| Barnea | 658.276 |
| | 859.9999999999999 |
| | None |
| Picholine | 249.334 |
| | 452.205 |
| | None |
| Koroneiki | 165.964 |
| | 430.881 |
| | None |
| Souri | 98.035 |
| | 346.42600000000004 |
| | None |
| Coratina | 497.421 |
*
Souri
*
*
*
*
Barnea
Souri
Coratina
Picholine
Koroneiki
Coratina
100 µm
