# Supplementary material for: High temperature environment reduces olive oil yield and quality
Source: PLoS One. 2020 Apr 23;15(4):e0231956. doi: 10.1371/journal.pone.0231956 (PMC7179852; doi:10.1371/journal.pone.0231956)
Supplement: S1 Table — (DOCX) [file pone.0231956.s006.docx]

**Supplementary Table 1:**

| **2016** | | | | | | |
| --- | --- | --- | --- | --- | --- | --- |
| **Month** | **Mean (Max (Tzuba (^o^C)))** | **Mean (Max (Tirat Zvi (^o^C)))** | **Max (Max (Tzuba (^o^C)))** | **Max (Max**  **(Tirat Zvi (^o^C)))** | **Mean (Min (Tzuba (^o^C)))** | **Mean (Min (Tirat Zvi (^o^C)))** |
| May | 27.7 | 36.6 | 40.6 | 46.4 | 14.1 | 17.8 |
| June | 32.8 | 41.8 | 37.8 | 46.4 | 18.4 | 21.7 |
| July | 32.8 | 42.1 | 35.2 | 44.8 | 18.5 | 24.4 |
| August | 32.6 | 42.9 | 36.1 | 46.3 | 20.0 | 25.2 |
| September | 30.8 | 40.7 | 35.2 | 43.9 | 17.8 | 21.9 |
| October | 29.4 | 37.2 | 34.4 | 43.9 | 16.8 | 18.4 |
| **2017** | | | | | | |
| May | 28.8 | 37.6 | 36.1 | 43.9 | 14.8 | 18.0 |
| June | 30.2 | 39.3 | 33.6 | 43.9 | 16.6 | 20.9 |
| July | 34.1 | 41.6 | 37.8 | 45.8 | 20.1 | 25.1 |
| August | 33.8 | 39.1 | 36.1 | 41.9 | 18.6 | 24.5 |
| September | 31.4 | 39.2 | 36.9 | 43.9 | 18.5 | 22.7 |
| October | 27.3 | 33.7 | 35.2 | 40.1 | 15.5 | 17.8 |
| November |  | 26.2 |  | 31.9 |  | 13.4 |
| December |  | 22.7 |  | 26.3 |  | 8.9 |
