# Supplementary material for: High temperature environment reduces olive oil yield and quality
Source: PLoS One. 2020 Apr 23;15(4):e0231956. doi: 10.1371/journal.pone.0231956 (PMC7179852; doi:10.1371/journal.pone.0231956)
Supplement: S2 Table — (DOCX) [file pone.0231956.s007.docx]

**Supplementary Table 2:**

|  |  | Mean humidity (%) | | Mean wind speed (m/s) | |
| --- | --- | --- | --- | --- | --- |
| Year | Month | Tirat Zvi | Tzuba | Tirat Zvi | Tzuba |
| 2016 | May | 50.2 | 54.9 | 3.5 | 3.1 |
| 2016 | June | 45.7 | 46.6 | 3.6 | 2.9 |
| 2016 | July | 54.0 | 63.1 | 3.6 | 2.9 |
| 2016 | August | 55.9 | 72.4 | 3.9 | 3.0 |
| 2016 | September | 54.8 | 66.4 | 3.3 | 2.7 |
| 2016 | October | 54.3 | 58.5 | 2.8 | 2.6 |
| 2016 | November | 45.2 | 48.2 | 2.6 | 4.3 |
|  |  |  |  |  |  |
| 2017 | May | 48.6 | 50.7 | 3.4 | 2.8 |
| 2017 | June | 51.8 | 57.5 | 3.6 | 2.6 |
| 2017 | July | 50.8 | 56.8 | 3.7 | 2.5 |
| 2017 | August | 54.8 | 69.0 | 3.6 | 2.4 |
| 2017 | September | 55.5 | 68.4 | 3.2 | 2.5 |
| 2017 | October | 53.5 | 66.1 | 2.7 | 2.4 |
| 2017 | November | 59.1 | 68.1 | 2.3 | 2.5 |
|  |  |  |  |  |  |
| Year | Location | Date | Daily rainful (mm) |  |  |
|  |  |  |  |  |  |
| 2016 | Tirat Zvi | May-05 | 2.4 |  |  |
| 2016 | Tirat Zvi | Nov-01 | 4.6 |  |  |
| 2016 | Tzuba | May-23 | 0.3 |  |  |
| 2016 | Tzuba | May-24 | 0.2 |  |  |
| 2016 | Tzuba | May-28 | 0.2 |  |  |
| 2016 | Tzuba | Oct-27 | 0.3 |  |  |
| 2016 | Tzuba | Nov-01 | 5.9 |  |  |
| 2017 | Tirat Zvi | May-20 | 0.3 |  |  |
| 2017 | Tirat Zvi | Oct-09 | 3.4 |  |  |
| 2017 | Tirat Zvi | Oct-27 | 0.5 |  |  |
| 2017 | Tirat Zvi | Oct-28 | 0.1 |  |  |
| 2017 | Tirat Zvi | Oct-29 | 0.2 |  |  |
| 2017 | Tzuba | May-19 | 0.3 |  |  |
| 2017 | Tzuba | May-20 | 0.2 |  |  |
| 2017 | Tzuba | Oct-09 | 5.3 |  |  |
| 2017 | Tzuba | Oct-27 | 1.5 |  |  |
| 2017 | Tzuba | Oct-30 | 0.7 |  |  |
| 2017 | Tzuba | Nov-01 | 0.4 |  |  |
| 2017 | Tzuba | Nov-02 | 0.1 |  |  |
| 2017 | Tzuba | Nov-08 | 0.2 |  |  |
