# Supplementary material for: High temperature environment reduces olive oil yield and quality
Source: PLoS One. 2020 Apr 23;15(4):e0231956. doi: 10.1371/journal.pone.0231956 (PMC7179852; doi:10.1371/journal.pone.0231956)
Supplement: S3 Table — (DOCX) [file pone.0231956.s008.docx]

**Supplementary Table 3:**

|  | **Tzuba** | **Tirat Zvi** |
| --- | --- | --- |
| **Cultivar** | **2016** | |
| Barnea | 3.62 +/- 1.2 | 2.44 +/- 1.2 |
| Coratina | 2.05 +/- 1.2 | 2.72 +/- 1.2 |
| Koroneiki | 2.52 +/- 1.2 | 3.63 +/- 1.2 |
|  | **2017** | |
| Barnea | 3.71 +/- 0.3 | 2.65 +/- 0.3 |
| Coratina | 1.12 +/- 0.3 | 2.33 +/- 0.3 |
| Koroneiki | 2.22 +/- 0.3 | 3.1 +/- 0.3 |
| Picholine | 3.06 +/- 0.3 | 2.78 +/- 0.3 |
| Souri | 3.03 +/- 0.3 | 2.83 +/- 0.3 |
