# Supplementary material for: High temperature environment reduces olive oil yield and quality
Source: PLoS One. 2020 Apr 23;15(4):e0231956. doi: 10.1371/journal.pone.0231956 (PMC7179852; doi:10.1371/journal.pone.0231956)
Supplement: S4 Table — a., f. The probability of the effects of cultivar type and tree location and their interaction, on the various parameters measured in this study, at two sampling dates in each year, 164 DPA and at harvest in 2016 and 146 DPA and at harvest time in 2017. Significant effects (P<0.05) are in bold font. b-e, g-j. Tukey-Kramer test, ranking the differences in dry fruit weight and dry fruit oil content between locations in the tested cultivars in 2016 at 164 DPA (b-c) and at harvest (d-e) and in 2017, at 146 DPA (g-h) and at harvest (i-j). The values appearing in the tables b-e and g-j are the added value in performance at the MT site compared to the HT site, calculated as a proportion of the HT results. (DOCX) [file pone.0231956.s009.docx]

| **a.** |  | | **Prob F** | | | |  | |  | |  | |  |
| --- | --- | --- | --- | --- | --- | --- | --- | --- | --- | --- | --- | --- | --- |
| **Date** | **Factor** | | **Dry fruit weight** | | **Dry fruit oil** | |  | |  | |  | |  |
| 164 DPA | Cultivar | | **3.08X10^-30^** | | **0.029** | |  | |  | |  | |  |
|  | Location | | **5.75 X10^-20^** | | **5.25 X10^-17^** | |  | |  | |  | |  |
|  | Interaction | | **4.74 X10^-11^** | | **1.88 X10^-6^** | |  | |  | |  | |  |
| Harvest | Cultivar | | **6.87 X10^-33^** | | **8.77 X10^-8^** | |  | |  | |  | |  |
|  | Location | | **1.38 X10^-14^** | | **2.08 X10^-11^** | |  | |  | |  | |  |
|  | Interaction | | **1.17 X10^-6^** | | **2.33 X10^-7^** | |  | |  | |  | |  |
|  |  | |  | |  | |  | |  | |  | |  |
| **b.** |  | |  | |  | | **c.** | |  | |  | |  |
| **Dry fruit - 164 DPA** | | | | |  | | **Dry fruit oil - 164 DPA** | | | | | |  |
| **Cultivar** | **MT>HT (%)** | | **Difference** | |  | | **Cultivar** | | **MT>HT (%)** | | **Difference** | |  |
| Coratina | 1.33 | | A | |  | | Souri | | 1.51 | | A | |  |
| Koroneiki | 1.07 | | A | |  | | Koroneiki | | 0.93 | | A | |  |
| Souri | 0.98 | | A | |  | | Coratina | | 0.48 | | B | |  |
| Barnea | 0.18 | | B | |  | | Barnea | | 0.09 | | B | |  |
|  |  | |  | |  | |  | |  | |  | |  |
|  |  | |  | |  | |  | |  | |  | |  |
| **d. Dry fruit - Harvest** | | | | |  | | **e. Dry fruit oil - Harvest** | | | | | |  |
| **Cultivar** | **MT>HT (%)** | | **Difference** | |  | | **Cultivar** | | **MT>HT (%)** | | **Difference** | |  |
| Koroneiki | 1.62 | | A | |  | | Koroneiki | | 0.61 | | A | |  |
| Coratina | 1.26 | | A | |  | | Coratina | | 0.13 | | B | |  |
| Barnea | 0.22 | | B | |  | | Barnea | | 0.04 | | B | |  |
| **f.** |  | **Prob > F** | | | | | | | | | | | |
| **Date** | **Factor** | **Dry fruit weight** | | **Dry fruit oil** | | **Cell area** | | **Cells layers** | | **Oil drop area** | | **Oil drops density** | |
| 146 DPA | Cultivar | **2.21X10^-18^** | | 0.13 | | **1.12 X10^-28^** | | **8.13 X10^-12^** | | **8.00 X10^-72^** | | **0.0256** | |
|  | Location | **2.67 X10^-15^** | | **8.62 X10^-25^** | | **2.26 X10^-24^** | | **8.83 X10^-5^** | | **3.06 X10^-21^** | | 0.51 | |
|  | Interaction | **0.000218** | | **1.52 X10^-6^** | | **0.002426** | | 0.17 | | **2.76 X10^-14^** | | **0.0022** | |
| Harvest | Cultivar | **4.08 X10^-22^** | | **3.96 X10^-12^** | | **5.43 X10^-11^** | | **2.22 X10^-8^** | |  | |  | |
|  | Location | **2.63 X10^-14^** | | **0.03447** | | **7.45 X10^-20^** | | **0.465834** | |  | |  | |
|  | Interaction | **1.16 X10^-7^** | | **0.001615** | | **2.32 X10^-7^** | | **6.01 X10^-12^** | |  | |  | |
| **g.** |  |  | |  | | **h.** | |  | |  | |  | |
| **Dry fruit - 146 DPA** | | | |  | | **Dry fruit oil - 146 DPA** | | | | | |  | |
| **Cultivar** | **MT>HT (%)** | **Difference** | |  | | **Cultivar** | | **MT>HT (%)** | | **Difference** | |  | |
| Souri | 1.04 | A | |  | | Koroneiki | | 1.61 | | A | |  | |
| Barnea | 0.63 | AB | |  | | Souri | | 1.14 | | AB | |  | |
| Koroneiki | 0.62 | AB | |  | | Coratina | | 0.78 | | BC | |  | |
| Coratina | 0.49 | B | |  | | Picholine | | 0.62 | | BC | |  | |
| Picholine | 0.36 | B | |  | | Barnea | | 0.48 | | C | |  | |
| **i.** |  |  | |  | |  | |  | |  | |  | |
| **Dry fruit - Harvest** | | | |  | | **Dry fruit oil - Harvest** | | | | | |  | |
| **Cultivar** | **MT>HT (%)** | **Difference** | |  | | **Cultivar**  **j.** | | **MT>HT (%)** | | **Difference** | |  | |
| Souri | 1.24 | A | |  | | Koroneiki | | 0.30 | | A | |  | |
| Koroneiki | 0.6 | B | |  | | Souri | | 0.26 | | A | |  | |
| Picholine | 0.52 | B | |  | | Picholine | | 0.07 | | B | |  | |
| Coratina | 0.41 | B | |  | | Coratina | | 0.04 | | B | |  | |
| Barnea | 0.16 | C | |  | | Barnea | | -0.13 | | B | |  | |
