# Supplementary material for: High temperature environment reduces olive oil yield and quality
Source: PLoS One. 2020 Apr 23;15(4):e0231956. doi: 10.1371/journal.pone.0231956 (PMC7179852; doi:10.1371/journal.pone.0231956)
Supplement: S5 Table — Errors are confidence limits (α = 0.05). (DOCX) [file pone.0231956.s010.docx]

**Supplementary Table 5:**

**2016:**

|  |  | **Fruit weight (gr)** | | **% oil commercial** | | **Oil per fruit (g)** | |
| --- | --- | --- | --- | --- | --- | --- | --- |
| **Cultivar** | **DPA** | **Tirat Zvi** | **Tzuba** | **Tirat Zvi** | **Tzuba** | **Tirat Zvi** | **Tzuba** |
| **Barnea** | 67 | 1.04+/-0.08 | 1.28+/-0.07 | 0.57+/-2.49 | 0.59+/-3.52 | 0.01+/-0.11 | 0.01+/-0.16 |
|  | 96 | 1.63+/-0.21 | 1.78+/-0.16 | 3.44+/-1.57 | 4.94+/-1.58 | 0.06+/-0.07 | 0.09+/-0.07 |
|  | 123 | 1.79+/-0.22 | 2.48+/-0.22 | 5.13+/-2.03 | 11.57+/-2.03 | 0.09+/-0.09 | 0.28+/-0.09 |
|  | 164 | 2.83+/-0.26 | 3.19+/-0.27 | 19.42+/-1.57 | 20.92+/-2.02 | 0.38+/-0.07 | 0.65+/-0.09 |
|  | 216 | 3.98+/-0.28 | 4.31+/-0.28 | 17.22+/-1.11 | 18.95+/-1.24 | 0.66+/-0.05 | 0.8+/-0.05 |
| **Coratina** | 67 | 1.2+/-0.11 | 1.45+/-0.11 | 0.38+/-0.29 | 0.58+/-0.29 | 0+/-0 | 0.01+/-0 |
|  | 96 | 1.74+/-0.3 | 2.58+/-0.28 | 1.99+/-0.44 | 3.61+/-0.44 | 0.04+/-0.01 | 0.09+/-0.01 |
|  | 123 | 2.23+/-0.31 | 3.51+/-0.32 | 2.56+/-1.42 | 9.23+/-1.23 | 0.05+/-0.08 | 0.33+/-0.07 |
|  | 164 | 2.36+/-0.49 | 5.11+/-0.57 | 16.93+/-3.09 | 20.93+/-4.66 | 0.27+/-0.11 | 0.83+/-0.08 |
|  | 216 | 2.96+/-0.26 | 5.51+/-0.34 | 16.63+/-4.42 | 19.7+/-3.42 | 0.46+/-0.16 | 0.98+/-0.12 |
| **Koroneiki** | 67 | 0.55+/-0.04 | 0.52+/-0.04 | 0.59+/-2.29 | 1.23+/-2.28 | 0.01+/-0.02 | 0.01+/-0.02 |
|  | 96 | 0.64+/-0.09 | 0.8+/-0.1 | 1.63+/-1.45 | 4.22+/-1.45 | 0.01+/-0.01 | 0.03+/-0.01 |
|  | 123 | 0.67+/-0.13 | 0.92+/-0.14 | 3.75+/-2.29 | 10.94+/-1.87 | 0.02+/-0.02 | 0.1+/-0.02 |
|  | 164 | 0.76+/-0.12 | 1.43+/-0.12 | 9.44+/-1.87 | 21.41+/-1.87 | 0.05+/-0.02 | 0.24+/-0.02 |
|  | 216 | 0.85+/-0.09 | 1.76+/-0.12 | 8.67+/-1.62 | 21.19+/-1.62 | 0.07+/-0.01 | 0.32+/-0.01 |
| **Souri** | 67 | 0.96+/-0.13 | 1.06+/-0.15 | 0.51+/-0.48 | 0.86+/-0.48 | 0+/-0 | 0.01+/-0 |
|  | 96 | 1.17+/-0.32 | 1.42+/-0.22 | 2.09+/-1.09 | 4.33+/-1.09 | 0.03+/-0.01 | 0.06+/-0.01 |
|  | 123 | 1.9+/-0.32 | 2.59+/-0.32 | 3.3+/-2.5 | 9.47+/-2.5 | 0.06+/-0.08 | 0.24+/-0.08 |
|  | 164 | 2.01+/-0.47 | 3.91+/-0.51 | 20.44+/-3.21 | 35.89+/-3.56 | 0.38+/-0.1 | 0.8+/-0.1 |

**2017:**

|  | |  | | **Fruit weight (gr)** | | **% oil commercial** | | **Oil per fruit (g)** | |
| --- | --- | --- | --- | --- | --- | --- | --- | --- | --- |
| **Cultivar** | | **DPA** | **Tirat Zvi** | **Tzuba** | **Tirat Zvi** | **Tzuba** | **Tirat Zvi** | **Tzuba** |  |
| **Barnea** | | 30 | 0.21 +/- 0.15 | 0.2 +/- 0.15 |  |  |  |  |  |
|  |  | 50 | 0.75 +/- 0.09 | 0.67 +/- 0.15 | 0.54 | 0.99 | 0.00 | 0.01 |  |
|  |  | 83 | 1.25 +/- 0.15 | 1.67 +/- 0.15 | 1.6 +/- 0.79 | 2.53 +/- 0.79 | 0.02 +/- 0.03 | 0.04 +/- 0.03 |  |
|  |  | 104 | 1.47 +/- 0.15 | 1.83 +/- 0.15 | 4.65 +/- 0.79 | 5.8 +/- 0.79 | 0.07 +/- 0.03 | 0.11 +/- 0.03 |  |
|  |  | 146 | 1.9 +/- 0.15 | 2.83 +/- 0.15 | 7.99 +/- 0.79 | 12.68 +/- 0.79 | 0.15 +/- 0.03 | 0.36 +/- 0.03 |  |
|  |  | 189 | 2.35 +/- 0.15 | 3.23 +/- 0.15 | 12.44 +/- 0.79 | 16 +/- 0.88 | 0.29 +/- 0.03 | 0.54 +/- 0.03 |  |
|  |  | 219 | 2.94 +/- 0.15 |  | 14.34 +/- 0.79 |  | 0.42 +/- 0.03 |  |  |
|  |  | 247 | 3.2 +/- 0.15 |  | 16.06 +/- 0.88 |  | 0.49 +/- 0.03 |  |  |
| **Coratina** | | 30 | 0.15 +/- 0.18 | 0.22 +/- 0.18 |  |  |  |  |  |
|  |  | 50 | 0.76 +/- 0.1 | 0.84 +/- 0.1 | 0.99 | 0.85 | 0.01 | 0.01 |  |
|  |  | 83 | 1.37 +/- 0.18 | 1.62 +/- 0.18 | 2.44 +/- 1.11 | 1.99 +/- 1.11 | 0.03 +/- 0.05 | 0.03 +/- 0.05 |  |
|  |  | 104 | 1.53 +/- 0.18 | 1.89 +/- 0.18 | 5.1 +/- 1.11 | 5.74 +/- 1.11 | 0.08 +/- 0.05 | 0.11 +/- 0.05 |  |
|  |  | 146 | 1.81 +/- 0.18 | 2.82 +/- 0.18 | 7.96 +/- 1.11 | 13.37 +/- 1.11 | 0.14 +/- 0.05 | 0.38 +/- 0.05 |  |
|  |  | 189 | 2.14 +/- 0.18 | 3.44 +/- 0.18 | 13.54 +/- 1.11 | 18.5 +/- 1.11 | 0.29 +/- 0.05 | 0.64 +/- 0.05 |  |
|  |  | 219 | 2.31 +/- 0.18 |  | 13.54 +/- 1.11 |  | 0.32 +/- 0.05 |  |  |
|  |  | 247 | 2.76 +/- 0.18 |  | 18.17 +/- 1.11 |  | 0.5 +/- 0.05 |  |  |
| **Koroneiki** | | 30 | 0.09 +/- 0.06 | 0.13 +/- 0.06 |  |  |  |  |  |
|  |  | 50 | 0.4 +/- 0.04 | 0.36 +/- 0.06 | 0.77 | 1.97 | 0.00 | 0.01 |  |
|  |  | 83 | 0.56 +/- 0.06 | 0.55 +/- 0.06 | 1.77 +/- 1.3 | 3.98 +/- 1.3 | 0.01 +/- 0.01 | 0.02 +/- 0.01 |  |
|  |  | 104 | 0.64 +/- 0.06 | 0.66 +/- 0.06 | 3.98 +/- 1.3 | 6.57 +/- 1.3 | 0.03 +/- 0.01 | 0.04 +/- 0.01 |  |
|  |  | 146 | 0.77 +/- 0.06 | 1.33 +/- 0.06 | 5.4 +/- 1.3 | 13.02 +/- 1.3 | 0.04 +/- 0.01 | 0.17 +/- 0.01 |  |
|  |  | 189 | 0.84 +/- 0.06 | 1.44 +/- 0.08 | 5.94 +/- 1.3 | 13.6 +/- 1.68 | 0.05 +/- 0.01 | 0.19 +/- 0.02 |  |
|  |  | 206 | 0.87 +/- 0.06 |  | 8.04 +/- 1.3 |  | 0.07 +/- 0.01 |  |  |
| **Picholine** | | 30 | 0.15 +/- 0.27 | 0.12 +/- 0.27 |  |  |  |  |  |
|  |  | 50 | 0.9 +/- 0.16 | 0.89 +/- 0.27 | 0.19 | 0.78 | 0.00 | 0.01 |  |
|  |  | 83 | 1.9 +/- 0.27 | 2.42 +/- 0.27 | 1.1 +/- 0.6 | 1.89 +/- 0.6 | 0.02 +/- 0.03 | 0.05 +/- 0.03 |  |
|  |  | 104 | 3.17 +/- 0.27 | 3.37 +/- 0.27 | 3.5 +/- 0.6 | 5.43 +/- 0.6 | 0.11 +/- 0.03 | 0.18 +/- 0.03 |  |
|  |  | 146 | 3.38 +/- 0.27 | 4.1 +/- 0.31 | 6.16 +/- 0.6 | 10 +/- 0.6 | 0.21 +/- 0.03 | 0.41 +/- 0.04 |  |
|  |  | 189 | 4.08 +/- 0.27 | 4.97 +/- 0.27 | 7.45 +/- 0.6 | 11.52 +/- 0.67 | 0.3 +/- 0.03 | 0.54 +/- 0.03 |  |
|  |  | 206 | 4.82 +/- 0.27 |  | 8.19 +/- 0.6 |  | 0.39 +/- 0.03 |  |  |
| **Souri** | | 30 | 0.09 +/- 0.17 | 0.17 +/- 0.17 |  |  |  |  |  |
|  |  | 50 | 0.64 +/- 0.1 | 0.8 +/- 0.12 | 1.00 | 0.78 | 0.01 | 0.01 |  |
|  |  | 83 | 1.44 +/- 0.17 | 1.71 +/- 0.17 | 1.13 +/- 1.16 | 1.31 +/- 1.16 | 0.02 +/- 0.03 | 0.02 +/- 0.03 |  |
|  |  | 104 | 1.65 +/- 0.17 | 2.23 +/- 0.17 | 3.27 +/- 1.16 | 6.52 +/- 1.16 | 0.05 +/- 0.03 | 0.15 +/- 0.03 |  |
|  |  | 146 | 1.64 +/- 0.17 | 3.1 +/- 0.17 | 6.62 +/- 1.16 | 13.76 +/- 1.49 | 0.11 +/- 0.03 | 0.46 +/- 0.03 |  |
|  |  | 189 | 1.98 +/- 0.17 | 4.69 +/- 0.17 | 10.31 +/- 1.16 | 13.94 +/- 1.29 | 0.2 +/- 0.03 | 0.7 +/- 0.04 |  |
|  |  | 206 | 2.24 +/- 0.17 |  | 10.45 +/- 1.16 |  | 0.23 +/- 0.03 |  |  |
