# Supplementary material for: High temperature environment reduces olive oil yield and quality
Source: PLoS One. 2020 Apr 23;15(4):e0231956. doi: 10.1371/journal.pone.0231956 (PMC7179852; doi:10.1371/journal.pone.0231956)
Supplement: S6 Table — The monthly gain of dry fruit weight and dry fruit oil percentatge for each cultivar, at each site for both years and was correlated to the temperatures data for each month (average daily maximum temp.–Tmax; average daily temp.–Tmean; average daily minimum temp.–Tmin). Correlation coefficient was calculated for all cultivars or for heat sensitive cultivars only. Significant correlation coefficients are highlighted in red font. (DOCX) [file pone.0231956.s011.docx]

| **All cultivars** | | | |
| --- | --- | --- | --- |
| **Variable** | **Variable** | **Correlation** | **P value** |
| Dry fruit weight | Tmax | -0.346 | 0.0016 |
| Dry fruit weight | Tmin | -0.2782 | 0.0119 |
| Dry fruit weight | Tmean | -0.3366 | 0.0021 |
| Dry fruit oil percentage | Tmax | -0.1827 | 0.1245 |
| Dry fruit oil percentage | Tmin | -0.0897 | 0.4538 |
| Dry fruit oil percentage | Tmean | -0.1222 | 0.3067 |
| **Only sensitive cultivars** | | | |
| **Variable** | **Variable** | **Correlation** | **P value** |
| Dry fruit weight | Tmax | -0.4356 | 0.0004 |
| Dry fruit weight | Tmin | -0.362 | 0.0038 |
| Dry fruit weight | Tmean | -0.4195 | 0.0007 |
| Dry fruit oil percentage | Tmax | -0.4015 | 0.0309 |
| Dry fruit oil percentage | Tmin | -0.2414 | 0.2072 |
| Dry fruit oil percentage | Tmean | -0.3218 | 0.0887 |
