# Supplementary material for: High temperature environment reduces olive oil yield and quality
Source: PLoS One. 2020 Apr 23;15(4):e0231956. doi: 10.1371/journal.pone.0231956 (PMC7179852; doi:10.1371/journal.pone.0231956)
Supplement: S7 Table — (DOCX) [file pone.0231956.s012.docx]

**Supplementary Table 7:**

**2016:**

|  | **Barnea** | | **Koroneiki** | | **Coratina** | |
| --- | --- | --- | --- | --- | --- | --- |
| **FA** | **Tirat Zvi** | **Tzuba** | **Tirat Zvi** | **Tzuba** | **Tirat Zvi** | **Tzuba** |
| **Palmitic acid** | 15.44 | 14.22 | 16.34 | 14.25 | 15.90 | 12.95 |
| **Palmitoleic acid** | 1.26 | 1.11 | 1.52 | 1.30 | 0.73 | 0.50 |
| **Margaric acid** | 0.05 | 0.00 | 0.05 | 0.00 | 0.05 | 0.00 |
| **Margaroleic acid** | 0.07 | 0.07 | 0.07 | 0.08 | 0.06 | 0.06 |
| **Oleic acid** | 55.52 | 63.06 | 66.67 | 70.77 | 62.75 | 69.28 |
| **Linoleic acid** | 23.34 | 17.45 | 10.11 | 9.28 | 15.63 | 12.70 |
| **Linolenic acid** | 0.42 | 0.37 | 0.55 | 0.43 | 0.52 | 0.44 |
| **Arachidic acid** | 0.75 | 0.70 | 1.19 | 0.80 | 0.99 | 0.80 |
| **Eicosenoic acid** | 0.23 | 0.22 | 0.34 | 0.30 | 0.42 | 0.47 |
| **Docosanoic acid** | 0.11 | 0.11 | 0.17 | 0.13 | 0.13 | 0.12 |
| **Stearic acid** | 2.33 | 2.20 | 2.32 | 2.16 | 2.25 | 2.03 |

**2017:**

|  | **Barnea** | | **Picholine** | | **Koroneiki** | | **Souri** | | **Coratina** | |
| --- | --- | --- | --- | --- | --- | --- | --- | --- | --- | --- |
| **FA** | **Tirat Zvi** | **Tzuba** | **Tirat Zvi** | **Tzuba** | **Tirat Zvi** | **Tzuba** | **Tirat Zvi** | **Tzuba** | **Tirat Zvi** | **Tzuba** |
| **Palmitic acid** | 15.02 | 13.73 | 21.62 | 18.67 | 16.26 | 14.12 | 14.41 | 13.46 | 14.69 | 11.94 |
| **Palmitoleic acid** | 0.94 | 0.89 | 2.18 | 1.85 | 1.72 | 1.24 | 0.91 | 0.64 | 0.67 | 0.46 |
| **Margaric acid** | 0.05 | 0.05 | 0.04 | 0.04 | 0.06 | 0.04 | 0.18 | 0.16 | 0.05 | 0.04 |
| **Stearic acid** | 2.77 | 2.69 | 2.41 | 2.32 | 2.54 | 2.27 | 4.31 | 3.44 | 2.46 | 2.31 |
| **Oleic acid** | 64.37 | 69.12 | 51.80 | 60.42 | 67.09 | 73.76 | 64.87 | 68.62 | 68.99 | 75.77 |
| **Linoleic acid** | 14.60 | 11.41 | 18.21 | 13.54 | 9.36 | 6.37 | 12.55 | 11.05 | 10.68 | 7.14 |
| **Linolenic acid** | 0.73 | 0.64 | 0.92 | 0.64 | 1.30 | 0.70 | 0.84 | 0.62 | 0.85 | 0.61 |
| **Arachidic acid** | 0.49 | 0.44 | 0.48 | 0.40 | 0.57 | 0.42 | 0.65 | 0.53 | 0.50 | 0.42 |
| **Eicosenoic acid** | 0.24 | 0.23 | 0.20 | 0.21 | 0.32 | 0.28 | 0.29 | 0.30 | 0.40 | 0.40 |
| **Docosanoic acid** | 0.13 | 0.12 | 0.13 | 0.11 | 0.18 | 0.13 | 0.16 | 0.14 | 0.13 | 0.11 |
| **Squalene** | 0.66 | 0.70 | 2.03 | 1.79 | 0.61 | 0.66 | 0.84 | 1.05 | 0.58 | 0.79 |
