# Supplementary material for: High temperature environment reduces olive oil yield and quality
Source: PLoS One. 2020 Apr 23;15(4):e0231956. doi: 10.1371/journal.pone.0231956 (PMC7179852; doi:10.1371/journal.pone.0231956)
Supplement: Attachment — Submitted filename: Editor and Reviewers comments.docx [file pone.0231956.s013.docx]

Before sending a manuscript or revised manuscript for review I need to ensure that it meets the publication criteria of PLOS ONE. One of the criteria is that ‘Experiments, statistics, and other analyses are performed to a high technical standard and are described in sufficient detail.’

The revised description of the statistical analysis has sufficient detail, however it suggests an error has been made in the use of the statistics:

“The differences between locations, dates and cultivars in the various parameters tested, such as fresh and dry fruit weight, oil concentration, amount of oil per fruit, cell size, oil droplet size etc. were subjected to multivariate analysis of variance (MANOVA) with three fixed factors (sample date, location and cultivar). Since in all analyses, interaction between the three factors was significant, we used one-way analysis of variance (ANOVA) and tested the differences between locations and dates in each cultivar separately, with a Tukey-Kramer test using JMP software.”

This experiment included multiple independent variables (three factors). MANOVA is used to assess the response of multiple dependant variables, not to assess the effect of multiple independent variables.

The approach you have described should use a three-way ANOVA or similar. Significant interactions in the independent variables in not a reason to switch to one-way ANOVA. In the case that all three independent variables have significant effects, the effects and their interactions need to be considered together.

This fundamental error in the analysis needs to be fixed in the methods section and also in the results and discussion sections, before the manuscript could be considered for publication.

If an appropriate analysis can be completed, you are welcome to resubmit a completely revised manuscript at a later date.

Response: Guided by your recommendations, I deepened the statistical analyses and integrated the detailed version into the manuscript.

This manuscript contains a considerable amount of new information on a very relevant topic. However, this revised version is still confusing in how the experiment is descripted and analysed. The experiment is essentially a genotype x environment field trial, partially repeated over two years. This type of experiment is very common and there are statistical conventions regarding how it should be presented. The authors need to place this manuscript in that context.
I agree with Reviewer 1 and 2 that the manuscript needs to be substantially shortened and reorganised. I would expect a revised manuscript to be at least half the length (approx. 6000 words). For instance:
Introduction- is well written, no need to repeat his content in the discussion.
 
Results- describe the abiotic conditions once at the beginning- don’t repeat for each parameter tested. The figures could also be more focused (see Reviewer 2 comments)
 
Methods- describe the experimental design once at the beginning- only. In the current manuscript the experimental design in not clear.
 
Discussion- needs to be shortened by at least half, remove repetition of content from results and introduction.
Done.
Reviewer 2 questions weather this experiment can really be described as testing heat stress or whether it is actually a comparison of two contrasting environments. This is a valid consideration, particularly as many of the parameters measured have little direct relation to heat tolerance, but are interesting harvest quality parameters. As well as making adjustments to the wording in the title, introduction and discussion, the authors need to make clear in the methods and abstract how long the stress period was (how long the plants were transferred to the different sites).

I would add to this that the real focus should be on comparing the response of the five genotypes, rather than trying to quantify the precise conditions in the two sites.

 In addition to the points above, I am not convinced of the statistical approach (see review 2 comments). The decision not to analyse the data from all the cultivars is questionable- consider doing this in the revised manuscript. The description of the statistics as a one-way ANOVA seems to be incorrect- the experiment involves two locations, and multiple cultivars (as well as two years), this cannot validly be tested with a one-way ANOVA. The statistical approach in the revised manuscript must be improved for this work to fulfil the criteria for publication in PLOS-ONE.

Done.
6. Review Comments to the Author

Please use the space provided to explain your answers to the questions above. You may also include additional comments for the author, including concerns about dual publication, research ethics, or publication ethics. (Please upload your review as an attachment if it exceeds 20,000 characters)

Reviewer #1: The revised version of the manuscript entitled “Heat stress reduces olive oil yield and quality” has been improved significantly but some important concerns remain. The length of the manuscript is excessive and some parts are difficult to understand due to the elevated number of cultivars/locations/components evaluated.

1. Please, consider adding in Abstract and Conclusions some numerical results (for example temperature thresholds)

Response: Added.
2. Result section is too long. The results must be shown clearly without a long introduction (for example, lines 395-402) or must be moved to Material and Method section (Lines 355-384). Some parts are difficult to follow are excessive information is provided.

Response: Lines 395-402 were deleted. Lines 355-384 belong to the results section because monitoring the environmental conditions at each of the experimental sites was as much an integral part of the experiment as the resulting development of the fruit during the two seasons the experiment was carried out.

Excessive information was reduced.
3. The length of the Discussion section is excessive. Authors have added numerous relevant publications but, in some cases, an excessive description of these studies has been detected (for example in Lines 765-773). The discussion section must compare and evaluate the results obtained in the study with other previous studies. The description of other studies must be avoided.

Response: Discussion was shortened and the comparison with previous publications was improved.
4. The small number of years considered in the study (specially for some cultivars) must be evaluated in the discussion section.

Response: This appears in the conclusion.
5. The text contains numerous words indicating suppositions. Thus, terms as “similar”, “perhaps” or “may be” must be avoided. All the conclusions provided in the manuscript must be funded in solid results. If uncertainty is detected, a study evaluating the causes of this, is required.

Response: These terms were deleted in most cases.

Specific comments:

6. Stomatal closure in olive is related to crop water status more than weather conditions. Thus, under high temperatures and low air humidity olive tree close stomas to reduce the water losses by transpiration. This is common during summer and it is the base for controlled deficit irrigation strategies. Please consider modifying the text in Lines 664-666.

Response: Deleted.
7. The study indicated in Lines 678-681 is not finally cited.

Response: Added
8. In Figure 2, please indicate with a symbol which results are statistically significant and which are not different.

Response: The error bars are confidence limits (α=0.05). Meaning, error bars that do not overlap represent significant differences. I added this in the figure legend.
9. Please, define “temperature differences” and avoid sentences as “However, temperature differences between the two locations were different” (Line 801)

Response: Deleted.
10. Climate change cannot be identified considering “last several years” (Line 841-843). Please, consider modifying these sentences.

Response: Deleted.

Reviewer #2: I have reviewed the revised version of the manuscript “Heat stress reduces olive oil yield and quality”. The author have made several changes, including a better revision of the literature, which is appreciated but still has been kept the Heat stress focus that it is not correct and I strongly suggest to be modified.
A heat stress is normally use as an indication of a relatively short term response to temperature above a certain threshold during a period of time. Normally it is related to maximum temperatures that are abnormally high and that are associated to a heat wave (natural or artificially simulated). On the other hand, adaptation to a high temperature environment is normally used for a longer term response and is associated with an environmental situation, location, condition in which mean temperature is normally high. This implies that not all temperatures along the day and along a period of time will necessarily be stressful and above a certain threshold. This is related with the physiological responses that will be generated: immediate (i.e., short term response) or acclimation response (i.e., long term response) in the second case to a prolonged temperature exposition. With global warming it is expected both phenomena: an increase in the mean temperature and an increase in the frequency of heat waves. A good example of the study of both phenomena can be found in grapes in which using the same general set up Soar et al. (2010) study the heat stress response to a simulated heat wave while Sadras et al. (2012) studied the prolonged response to elevated temperature. In both cases the response variables were similar but involved different physiological mechanism. Then, in the present study in olive it was study a prolonged exposition to high temperature environment with possible higher frequency of heat stress episodes (i.e., temperatures above a certain threshold) in Tirat Zvi than in Tzuba. If the author would be interested in evaluated the impact of a heat stress at least they should have had to analyzed the heat load (e.g., Barlow et al. 2015). However, there is a problem for this calculation due to a relatively low frequency of data acquisition (every two hours) for this kind of calculations. An additional problem in olive it is that there is not enough information that help defining cardinal temperature for different processes (like oil synthesis) to be used in the heat load calculations. Although Garcia-Inza et al. (2018) analyzed the different dimensions of temperature (Tmax, Tmin, T mean, thermal amplitude) they still were analyzing the impact of prolonged high temperature exposition and not heat stress because temperature was changing along the season and they used average values.
Based on the previous paragraph comments, I think that the title of the article, the introduction of the manuscript and, in consequence the rest of the article should be change to reflect what was studied: the dynamic of fruit growth, oil concentration, and the final obtain oil quality in two contrasting environment. This is similar to previous works, particularly Rondanini et al. (2014) and Trentacoste et al (2012). Of course it is important and very interesting that the Tirat Zvi has very high temperatures and in this respect the most important contribution of the present study should be that they are extending the range of conditions in which fruit growth and oil quality have been previously evaluated. However, this comparison and that with the manipulative experiments from García-Inza is lacking along the discussion. The articles are mention but a deeper comparison of the studies is lacking.
Lastly, I found this version of the manuscript long (particularly the discussion), and in parts repetitive. I think the data are interesting and the manuscript has potential but needs to improve the writing and presentation. Following I am including a detail list of comments to be considered.

1) Change title deleting the “Heat stress” mention

Response: Changed.
2) Line 37 Too strong statement. Change IDENTICAL for SIMILAR. Even when you grew plants in pot and under similar conditions olive never is identical one plant from the other.

Response: Changed.

3) Introduction: the first paragraph in focus on heat stress, the references to real heat stress studies, and that should be change in accordance to what it was study in the present work. Also the introduction is long and focusing it and shortening would be appreciated by the readers

Response: Changed.

4) Line 74: There mixing references. Some of the mention studies are heat stress studies and others analyzed the responses to high temperature.

Response: We sorted the references according to the analysis they performed.

5) Lines 91-94. I think that the later studies of Hammami and Rapoport are more critical for you than that from Rallo-Rapoport and should be considered here and in the discussion
Response: Hammami et al. (2011) was added.
6) Line 123. The mention article (number 25) is a poorly accessible article and reading the abstract it is not related to the impact of environment on fatty acid composition. Deleted and change for something better
Response: An appropriate reference was added.
7) Lines 123-125: None of these works study heat stress but the relation of mean, maximum, minimum temperatures between regions and/or years or manipulating temperature but for long periods of time and not a short term stressful event. Also, Trentacoste did not study fatty acid composition
Response: Corrected.
8) Line 189. This amount of water seems TOO BiG for the size of the pots and potentially that of the trees. Which was the leaf area per tree? Which was the estimated crop evapotranspiration? This is an important issue to address because in the discussion it is mention that possible differences in irrigation frequency could have generated differences in canopy temperature that could affect fruit growth. On the other hand, if the amount of water was too high it can also be possible that plants have been exposed to water excess during certain periods of time. An estimate of ETc would help understanding this
Response: Olive trees of the same age, in commercial orchards, are usually irrigated with approximately 70 liters per day per tree in Israel. Leaf area was about 3 square meters per tree. Therefore, 12 liters per day per tree works out to 4 m^3^ per dunam (1000 m^2^), which is really not so high. ETc is estimated in these location as 0.3-0.6 during the year (depending on location and time of year). In any case, the planting pots were all equipped with holes in the bottom for escape of excess water. The pots were visited regularly, and there were no signs of excess water.

9) Line 209 how many weeks after flowering the plants were move to the two sites. Comment. This part of the materials and methods needs a reorganization to clearly explain the general procedure in relation to the plant phenological stage.
Response: We added the require data.
10) Line 218 Change Mesocarp by mesocarp
Response: Changed.
11) Lines 168 and 171,”(31°59'33.5"N 34°49'10.0"E; 42 m above sea level)”. I suggest deleting the seconds in the latitudes and longitudes
Response: Deleted.
12) In the same paragraph I suggest to include an easy to follow name for the locations like: high temperature site (HT) and moderate temperature site (MT). The names of the two locations have certain repetitions of letter (T Z) that makes it hard to remember which is which for a reader outside Israel.
Response: The two locations were named "MT" and "HT".
13) Lines 343-346 If you are mentioning that location and cultivar were analyzed together then you do not have a ONE way ANOVA. Also, if you are analyzing successive dates, that needs to be analyzed by repeated measurements
Response: We changed this paragraph and wrote: "The differences between locations, dates and cultivars, in the various parameters tested, such as fruit weight, oil concentration, amount of oil per fruit, cell size, oil droplet size etc. were subjected to multivariate analysis of variance (MANOVA) with three fixed factors (sample date, location and cultivar). Since in all analyses, interaction between the three factors was significant, we used one-way analysis of variance (ANOVA) and tested the differences between locations and dates in each cultivar separately, with a Tukey-Kramer test using JMP software."
14) Results. There is something very important missing here. Fruit load; (# fruit/ leaf area or yield/ leaf area o canopy volume) is something that needs to be included before talking about fruit weight or oil concentration. Particularly in these trees that are rather small, a little change in fruit number could generate an important differences in fruit load or source:sink ratio and that affects fruit size, oil concentration, pulp:pit, maturity index and phenolics as much or more than temperature
Response: We accept the truth of this comment. Although we analyzed many parameters, we missed some. Therefore, we added in the experimental design session the following sentences: "We did not measure fruit load, although this could have an effect on fruit weight as well as on oil concentration. However, plants were randomly chosen to be placed at the two locations and therefore we believe that the average fruit load was similar at the HT and the MT sites."
15) Fig. 1 This graph repeats information of fig 2 in other way. I recommend you either leave this with daily values and delete the fig 2 with monthly values or have that as fig 1 and delete this
Response: Done.
16) Fig. 2 This figure needs to be improve. As it is currently presented it needs two pages to compare the two cultivars. I recommend: a) delete the temperature part (in fact it is repeated in part a and b and in Fig 1 with another frequency; b) have scales similar between years and cultivars for a given variable (e.g., % oil goes from 0-20% in one year and from 0-25% the other; c) include the information for the 5 genotypes and do not limited to 2 cultivars; d) have the data of fruit weigh and oil% on the same basis. That is fruit dry weight and oil% estimated for dry weigh (this would be better than fresh weigh in which differences in temperature could generated differences in relative water content of the fruits); e) delete the oil per fruit because in fact it comes from multiplying fruit dry weight by % oil. In this way you reduce the space but extending the presented information to a larger group of cultivars. In fact you could divided in a fruit dry weight graph and an oil% graph, with 10 panels each (2 years x two cultivars)

Response: Done.
17) Fig. 3 I think that this graph would be way better if: 1) delete the microscopic images that do not contribute much (you are not marking anything particularly there, the quality is not great and makes the graph very big); 2) divide in two graph one for cell layer and other for cell area. 3) delete fruit weigh because it is already included in the previous graph; 4) include the five cultivar which would be more interesting
Response: Done.
18) Fig 5 . I have similar comments as fig 3: 1) oil % have been already presented so there is no point in presented it again; 2) I do not think that the pictures bring anything new. Also the quality is not that good so I recommend deleting the pictures but to include the information of number of oil drops and area for the five cultivars.
Response: Done.
19) Fig. 7 this figure needs to be improve: 1) include error bars as a measurement of dispersion of data; 2) the axis needs to be similar between years for a given variable; 3) reduce the number of ticks!!. For polyphenols you have 9 ticks and numbers; and 4) increase the size of letters and numbers in axis and reduce the size for the axis name.
Response: 1) There are no error bars since at harvest, we had enough fruits for only one oil extraction. Therefore we do not have biological replicates. 2) We have improved graphic representation of the oleic acid content. However, since polyphenols level was dramatically different between years, the minimum and maximum axes must be different between years, in order to illustrate the differences between HT and MT. 3+4) Done.
20) Line 356 I recommend to evaluate the possibility of changing the names of the location in some way. Now both names start with T and there is also a Z in the name. For the reader not familiarized with these locations it is hard to remember which location is which.
Response: Done.
21) Line 371 it reads “several rainy days. “Do you want to say that or FEW rainy days?
Response: Changed.
22) Lines 394-489. This section is long and hard to follow. Reduce text and take into consideration the suggestions. One of the problems I found it is that it goes from one variable to the other and return to the first. Probably taking in consideration the suggested changes in the figures would help solving the problem
Response: Corrected.
23) Line 396 and other part. When talking about fruit weight it is important to indicate if you are referring to the dry or the fresh weigh. This also includes figure legends and axis title. If you are showing the results of fresh weight you would need to include the information of water content as Trentacoste et al. 2012
Response: Changed to dry weight.
24) Lines 395-428. The paragraph is very long. Also, the dynamics are strongly influenced by change in meteorology along the season and between seasons. Perhaps presenting the data as a function of thermal time as Trentacoste or Rondanini could give you a smother function. This would make comparisons with literature easier; something limited now
Response: Our study did not analyze the dynamics of the influence of high temperatures on olive fruit development along a yearly or seasonal axis. Instead we studied the cumulative effect of this environmental factor on several important parameters as they appeared at harvest time. Therefore, we would prefer to present it as it is (and as Trentacoste et al. did). We hope that with the changes made in this revision, comparisons with existing literature will be more straightforward.

25) Lines 442-443 “No massive fruit abscission was detected.” This is not clear for me. Are you talking to the event of normal flower/fruitlet abscission after pollination? The normally expected fruit set is 5-10% inflorescence to set at least one fruit. Was this value higher under your conditions? Why?
Or are you talking about that plants were transfer to Volcani before harvesting all fruits? Why did you kept fruits in the trees for that long then? why to transfer if you were expecting to compared locations? How keeping the fruit for so long affected return flowering?
Response: We located the plants at the HT and MT sites after fruit set. During the season, when the trees were in place at the HT and MT sites, there was no massive fruit abscission. However, these details were deleted, as suggested earlier.
26) Line 444. Correct this. Yield includes the number of fruit as one of the main components. So change the name or the calculations
Response: This part was deleted.
27) Line 496. Include the figure number you are talking about.
Response: Added.
28) Line 547 . “Oil drop density “ Change in the figure axis so both, text and figure are consistent
Response: Figure axis title changed.
29) Line 578. Include the figure number you are talking about.
Response: Added.
30) Lines 593-95 This is not clear. Are you talking about percentual points of oleic acid or percentage differences between locations?
Response: Corrected.
31) Line 604 . palmitic Acid (C16:0) and linoleic Acid (C18:2) in oil. No need of a capital letter for Acid
Response: Corrected.
32) Discussion. This needs substantial improvement. It is too long (12 pages), in part repetitive and lack a comparison with the literature that help to put your data in a more general response to temperature but with your important addition of including information at very high temperatures.
I am only including some comments but this section needs more than that.
Response: It was shortened to 10 pages and redacted. Corrections were made according to received comments and several more changes made to improve the discussion.
33) Line 641 “ However… time”. I am not that sure. If it is well know this statement needs a good citation.
Response: The sentence has been changed and reference was added.
34) 651 You are forgetting fruit number, the most critical yield component
Response: Added.
35) Lines 656-658 This does not make sense. First you say that Koroneiki is sensitive and the following phrase you say it is tolerant to high temperature
Response: The text reads that in contrast to 'Koroneiki', the 'Barnea' cultivar is tolerant to high temperatures.
36) Lines 662-667 This inclusion here cut the flow of the paragraph and you are not giving a clear message. The other point: did you do calculations to evaluate if the plants could have go under certain water stress that could have generated differences in temperature? This would not be difficult to do. My impression is that it is not the case and then the statement would make no sense.
Response: The sentence was deleted.
37) Lines 651-669 This paragraph seems to be devoted to the genotype differences. In this respect there are some cultivars that you studied and that have been evaluated before by Cecci, Lombardo, Mailer, Rondanini, Trentacoste (etc). Particularly for fatty acid composition there is a lot of information. It would be interesting to compare if the responses of the genotypes are different or not between studies. Again, making the point that you included a site with higher temperatures than the rest of the locations (although you need to do the comparisons of temperature to be sure that’s the case)
Response: Relevant points in comparable studies were analyzed.
38) The following paragraph needs to focus. There is not a clear message
Response: Done.
39) Lines 671-673 The data presented in the S5 table are not clear. You need to perform a correlation analyzes between average min temperature, and final oil% with all cultivars, years, and locations. As you have only two locations (i.e., two min temperature points) and two years it will be hard to have a good relation if considering each cultivar differently. I am suggesting you to try what Garcia-Inza did: compared maximum oil% with Tmean, T min and Tmax
Response: Done.
40) Lines 689-734 It is important that you talk about dry weight. Otherwise you could have differences in water content in the fruits and not biomass. That could be related to differences in fruit transpiration between locations.
This part is very disorganize and makes it difficult to follow. Why don’t you first talk about fruit dry weight, than cell number and lastly cell expansion?.
Response: Done.
41) lines 690-700 Note, having many works in olive in which temperature effect was evaluated on fruit weight, oil concentration and quality, it does not make sense citing apple which it is a very different crop, the environment in which it grows is normally quite cool, and that accumulates a fair amount of water... not oil. Very different physiology
Response: Deleted.
42) Lines 704-706 How many weeks after flowering were the treatments applied? Could you expect an influence of the different temperatures on cell number?? Compared this with the information from Hammami et al. 2011
Response: Done.
43) Lines around 709. Here you need to compare the results not with apple but with more recent works from Rapoport and Hammammi. It is hard to believe that so late in the season you had so much cell division. Could not it be an artifact of how cuts were done?. Please this is an important issue to analyze because Rapoport has several studies on the cell division and expansion dynamic of the different parts of the fruit and your results seems to contradict those. If that it is really the case a good argument would be needed.
Response: We don't think our results are artifacts. We included their findings and compare it to ours.
44) Line 718. I understand this but, how many weeks after full bloom did you move the plants? Also and VERY important, if you move it early in the season it is highly possible that you lost fruits. If that the case, the number of fruit would be controlling cell division strongly. Again you need information on fruit load.
Response: Transportation was carried out two weeks after full bloom and was done very carefully. No fruit drop was observed due to the trees moving. We added this information to the text.
45) Lines 788-790. I do not think you can say this with your data because have average temperatures and in this location there is great daily amplitude
Response: The entire paragraph was deleted.
46) Lines 820-823. I suggest deleting or modifying this phrase because you are not mentioning ALL the parameters related to chemical quality (e.g., sterols, tocopherols, wax...) so this needs to be modify to avoid saying something wrong.
Response: Done.
47) Lines 838 again, do the calculations: which was the expected ETc and how many mm did you water?. Also would be important to explain if there was any water excess.
Response: See the response to comment 8.
48) lines 841-844. This do not add anything importatnt. Delete
Response: Deleted.
49) lines 836-877. This is a VERY long paragraph. Please reduce and focus
Response: Done.
50) Lines 868-874. Delete. You are talking about fatty acid and again jump to oil concentration
Response: We moved this section to the relevant paragraph.
51) Revise the references. There format mistakes and also references incorrect
Response: Done.
